# Ambiguous definitions for baseline serum creatinine affect acute kidney diagnosis at the emergency department

**DOI:** 10.1186/s12882-021-02581-x

**Published:** 2021-11-08

**Authors:** Michael Niemantsverdriet, Meriem Khairoun, Ayman El Idrissi, Romy Koopsen, Imo Hoefer, Wouter van Solinge, Jan Willem Uffen, Domenico Bellomo, Wouter Tiel Groenestege, Karin Kaasjager, Saskia Haitjema

**Affiliations:** 1 Central Diagnostic Laboratory, University Medical Center Utrecht, Utrecht University, Room Number G03.551, UMC Utrecht, Heidelberglaan 100, Utrecht, 3584 CX The Netherlands; 2 SkylineDx, Lichtenauerlaan 40, Rotterdam, 3062 ME The Netherlands; 3 Department of Nephrology and Hypertension, University Medical Center Utrecht, Utrecht University, Heidelberglaan 100, Utrecht, 3584 CX The Netherlands; 4 Department of Internal Medicine, University Medical Center Utrecht, Utrecht University, Heidelberglaan 100, Utrecht, 3584 CX The Netherlands

**Keywords:** AKI, Creatinine, CKD-EPI, Electronic health records, Digital health

## Abstract

**Background:**

Acute kidney injury (AKI) incidence is increasing, however AKI is often missed at the emergency department (ED). AKI diagnosis depends on changes in kidney function by comparing a serum creatinine (SCr) measurement to a baseline value. However, it remains unclear to what extent different baseline values may affect AKI diagnosis at ED.

**Methods:**

Routine care data from ED visits between 2012 and 2019 were extracted from the Utrecht Patient Oriented Database. We evaluated baseline definitions with criteria from the RIFLE, AKIN and KDIGO guidelines. We evaluated four baseline SCr definitions (lowest, most recent, mean, median), as well as five different time windows (up to 365 days prior to ED visit) to select a baseline and compared this to the first measured SCr at ED. As an outcome, we assessed AKI prevalence at ED.

**Results:**

We included 47,373 ED visits with both SCr-ED and SCr-BL available. Of these, 46,100 visits had a SCr-BL from the − 365/− 7 days time window. Apart from the *lowest* value, AKI prevalence remained similar for the other definitions when varying the time window. The *lowest* value with the − 365/− 7 time window resulted in the highest prevalence (21.4%). Importantly, applying the guidelines with all criteria resulted in major differences in prevalence ranging from 5.9 to 24.0%.

**Conclusions:**

AKI prevalence varies with the use of different baseline definitions in ED patients. Clinicians, as well as researchers and developers of automatic diagnostic tools should take these considerations into account when aiming to diagnose AKI in clinical and research settings.

**Supplementary Information:**

The online version contains supplementary material available at 10.1186/s12882-021-02581-x.

## Background

Acute kidney injury (AKI) is the most common complication in hospitalized patients and is associated with high morbidity and mortality [1]. The incidence of AKI is increasing due to the growing incidence of risk factors, including cardiovascular disease, use of nephrotoxic medication and contrast-containing imaging [2]. As mild increases of serum creatinine (SCr) are associated with adverse outcome, it’s important to identify risk factors and to increase awareness of AKI in healthcare systems [3]. However, AKI is often missed, due to lack of awareness and knowledge on early recognition, prevention and management of AKI by physicians from different specialties [4]. Importantly, recent studies indicated that early detection of AKI improves short and long-term outcomes [5].

To improve awareness and promote early detection by health care providers, multiple guidelines have been developed, including *Risk, Injury, Failure, Loss and End-stage* (RIFLE), *Acute Kidney Injury Network* (AKIN) and *Kidney Disease Improving Global Outcomes* (KDIGO), as this may lead to initiation of early interventions, such as adequate fluid management, adjustment of medication dose and avoiding the use of specific nephrotoxins [6–8]. Overall, these guidelines postulate criteria for AKI diagnosis by detecting changes in endogenous surrogate markers of kidney function, such as SCr and urine output [9].

Growing use of electronic health records (EHR) and machine learning have provided a possibility to study large collections of real-world data and develop early detection systems for AKI [10]. Indeed, clinical decision support systems (CDS) have emerged as tools for initial assessment and identification of AKI patients in different settings [11]. These CDS make recommendations and risk stratifications based on the existing guidelines and best practices for AKI [12]. Understanding the specific definitions of the guidelines and their implications on AKI diagnosis is thus of utmost importance in both patient care and research.

In brief, the diagnostic criteria for AKI are based on a change between a current SCr and a previous SCr measurement known as ‘baseline’ in the preceding days or period. As kidney function of hospitalized patients is routinely monitored, SCr measurements during admission are often available to compare with the baseline value, usually defined as the first measurement during admission or at ED presentation. However, patients who visit the ED may lack SCr measurements from the pre-admission period, making the criteria less suitable for this setting. As a consequence, several studies have proposed multiple ways to define baseline using different values and time windows [13].

Multiple definitions have been used in literature to select a baseline value from the patient’s clinical history, such as the most recent value, the median value, the mean or the lowest value [14–16]. Depending on available data, researchers have applied varying lengths for the baseline time window and baseline definitions for AKI diagnosis [17]. The use of different definitions has been shown to affect AKI prevalence in hospitalized patients [13]. However, to which extent the use of various baseline definition and time windows influence AKI diagnosis in the ED has not been investigated.

Here, we systematically compared various baseline definitions using AKI criteria from the RIFLE, AKIN and KDIGO guidelines with several combinations of baseline time window and baseline definition to assess the effect on AKI diagnosis, using prevalence as an outcome, in a large cohort of ED patients from our center.

## Methods

### Study population

We performed a single center retrospective analysis, using routine care data from the University Medical Center Utrecht (UMCU), a large tertiary referral center in Utrecht, the Netherlands. All ED visits between 2012 and 2019 from adult patients over 18 years of age were included. Data was extracted from the Utrecht Patient Orientated Database (UPOD). In brief, UPOD is an infrastructure of relational databases comprising data on patient characteristics, hospital discharge diagnoses, medical procedures, medication orders and laboratory tests for all patients treated at the UMCU since 2004 [18].

From UPOD, for each ED visit we included patient’s age, gender and treating specialty. Additionally, all SCr measurements were extracted 365 days prior to ED visit, as well as the first measurement at ED. We defined the latter as the first measurement within 6 h after ED admission, as the majority of ED visits are either send home or admitted to the hospital within hours after ED admission (SCr-ED). ED visits with no SCr measurements 365 days prior to ED visit and a SCr-ED were excluded from this study. SCr was measured by isotope dilution mass spectrometry traceable enzymatic colorimetric assays (*Beckman Coulter, Brea, CA, USA and Siemens Healthcare Diagnostic Inc., NY, USA*). Estimated Glomerular Filtration Rate (eGFR) was calculated by the CKD-EPI formula [19]. Chronic Kidney Disease (CKD) was defined according to the KDIGO criteria based on eGFR.

### AKI criteria

To compare the effect of varying baseline definitions on AKI prevalence in the ED, we applied criteria from the RIFLE, AKIN and KDIGO guidelines. As RIFLE does not specify a baseline time window and KDIGO only specifies a time window of 7 days, we used a window of 365 days to define SCr-BL. We did not include the urine output criteria as this is not available in most hospitals. We defined four SCr criteria and three eGFR criteria applicable to the ED to determine AKI (Table 1) [15, 20]. Four of the seven criteria specify a baseline time window: two rely upon a window of 7 days (− 7 / 0 days) and two define a window of 1 year to a week prior ED visit (− 365 / -7 days). The remaining three criteria do not define a specific baseline value, but compute changes in the surrogate marker measurements from the 48 h prior to the ED visit. Each available measurement from this time window was compared as a baseline to the SCr-ED.

**Table 1:** Seven criteria for AKI diagnosis adapted from the RIFLE, AKIN and KDIGO guidelines

Criteria number	Criteria	RIFLE	AKIN	KDIGO
1	Rise of ≥26.5 SCr 48 h prior to ED admission		V	V
2	Relative increase of ≥1.5 SCr to baseline within 48 h prior to ED admission	V	V	V
3	Relative increase of ≥1.5 SCr to baseline within 7 days	V		V
4	Relative increase of ≥1.5 SCr to baseline within 1 year	V		V
5	Relative decrease of > 25% eGFR 48 h prior to ED admission	V		
6	Relative decrease of > 25% eGFR to baseline within 7 days	V		
7	Relative decrease of > 25% eGFR to baseline within 1 year	V		

Each criterion compares a specific value extracted from a baseline time window before emergency department (ED) with the value measured at the ED. “V” indicates that the criterion is part of the specific guideline. AKI is diagnosed when at least one of the criteria per guideline are met

### Analysis

As patients can have multiple SCr measurements within a baseline time window, we evaluated baseline definitions in terms of time window and value. This was done only for the four criteria who specify a baseline in the time window prior to ED visit. For each patient visit, we included all SCr measurements from the patient’s medical history from the baseline time window. Then, from this set of SCr measurements we defined baseline serum creatinine (SCr-BL) for each criterion by applying the four baseline values: median, mean, most recent and lowest.

Additionally, to further characterize the effect of the time window and baseline value combinations, we looked at the effect on AKI prevalence of a varying time window lengths. More specifically, we looked at the combinations of all the four baseline value options and five time windows ranging from − 365 days prior up to 7 days prior to ED presentation (− 365/− 7, − 270/− 7, − 180/− 7, − 90/− 7, − 45/− 7 days). For each of these 20 combinations we defined a SCr-BL.

After determining the SCr-BL for the four baseline criteria, we calculated the AKI prevalence for each individual criterion with the SCr-ED for every ED visit. In order to use the three eGFR criteria, we calculated the eGFR for each selected SCr value with the CKD-EPI formula. Subsequently, we compared the AKI prevalence between the three guidelines by computing the remaining three criteria who did not define a baseline time window. AKIN prevalence was only computed for patients with measurements in the 48 h prior to ED visit. Finally, we computed the AKI prevalence for each guideline. The first stage of AKI was deemed as having AKI for all analyses. Data are presented as means with standard deviations. All data pre-processing and analyses were performed using the R environment (version 3.6.1).

## Results

### Baseline characteristics

**Fig. 1:**
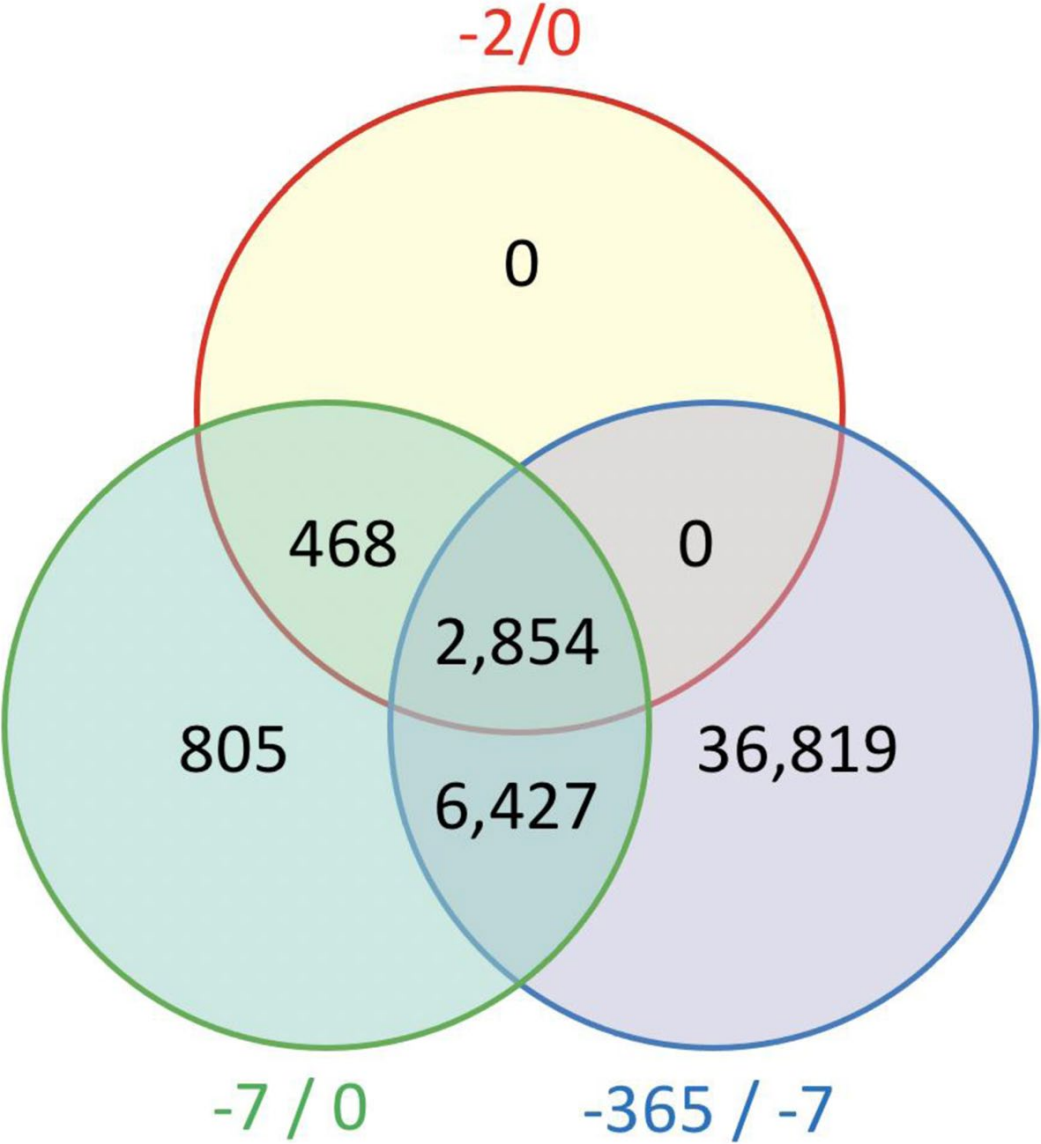
Number of visits with overlapping serum creatinine measurements from the three baseline time windows

We included 20,488 patients who visited our ED between 2012 and 2019, with both a SCr-ED and a SCr-BL available in their EHR, which corresponded to a total of 47,373 visits (Table 2). 54.9% of the patients had one ED visit (Supplementary Table 1). Most visits were made by men (53.5%), with an average age of 59.0 ± 16.8 and a mean SCr-ED of 108.3 ± 133.2. One third of the visits were patients that were subsequently admitted to the internal medicine department (32.3%). Of the 47,373 visits, 46,100 (97.3%) had at least one SCr measurement available within the − 365/− 7 baseline time window, 10,554 (22.3%) in the − 7/0 and 3322 (7.0%) in the − 2/0 window (not mutually exclusive) (Fig. 1).

**Table 2:** Baseline characteristics of all filtered emergency department visits

	*N* = 47,373 ED visits
Age, years mean (SD)	59.0 (16.8)
Male sex, count (%)	23,358 (53.5%)
Hospitalized, count (%)	29,633 (62.6%)
CKD category at ED presentation, % (N)	
G1	18,246 (38.5%)
G2	15,491 (32.7%)
G3a	5299 (11.2%)
G3b	3965 (8.4%)
G4	2746 (5.8%)
G5	1626 (3.4%)
ED specialty, count (%)	
Cardiology	9003 (19.0%)
Gastroenterology	2885 (6.1%)
Internal medicine	15,233 (32.2%)
Pulmonary disease	5358 (11.3%)
Nephrology	2267 (4.8%)
Neurology	5211 (11.0%)
Surgery	4710 (9.9%)
Urology	2165 (4.6%)
Other	541 (1.1%)
SCr-ED, μmol/L, mean (SD)	108.3 (113.2)
Baseline SCr measurements frequency in the previous 365 days before ED visit, count (%)	
1 SCr measurement	8710 (18.4%)
2 SCr measurements	5643 (11.9%)
3 SCr measurements	4249 (9.0%)
4 SCr measurements	3521 (7.4%)
5 SCr measurements	2792 (5.9%)
> 5 measurements	22,458 (46.9%)

Only emergency department visits with a serum creatinine measurement at emergency department visit and at least one baseline serum creatinine value were selected. Percentages reflect the percentage of the total number of visits. Emergency department specialty was defined as the first specialty the patient encountered during visit

### Lowest baseline value leads to the highest AKI prevalence

**Fig. 2:**
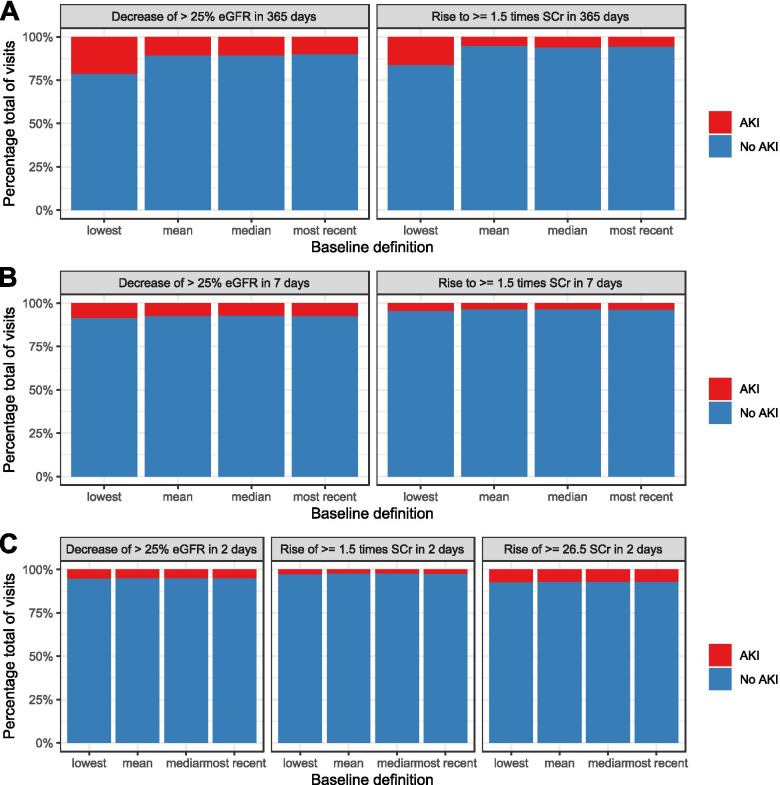
AKI prevalence at emergency department for all seven criteria with the four baseline values. Panel **A** shows the two − 365/− 7 criteria, panel **B** shows the two − 7/0 criteria and panel **C** shows the three − 2/0 criteria

We used four baseline definitions to calculate the delta for both surrogate markers for the − 365/− 7 and − 7/0 days time windows. We found that the lowest baseline definition resulted in the highest mean delta for both surrogate markers regardless of time window (Supplemental Table 2, Supplemental Figs. 1 and 2). Furthermore, we found a similar effect for the lowest value regardless of time window when we applied the seven criteria on our data (Fig. 2). The eGFR criteria resulted in a higher AKI prevalence compared to the SCr criteria (Supplementary Data Table 2). In particular, using the lowest value for both the SCr and the eGFR criteria for the − 365/− 7 baseline time window resulted in the highest relative AKI prevalence, 15.8 and 21.4% respectively (Supplemental Table 3).

### Reducing the baseline time window width decreases AKI prevalence

**Fig. 3:**
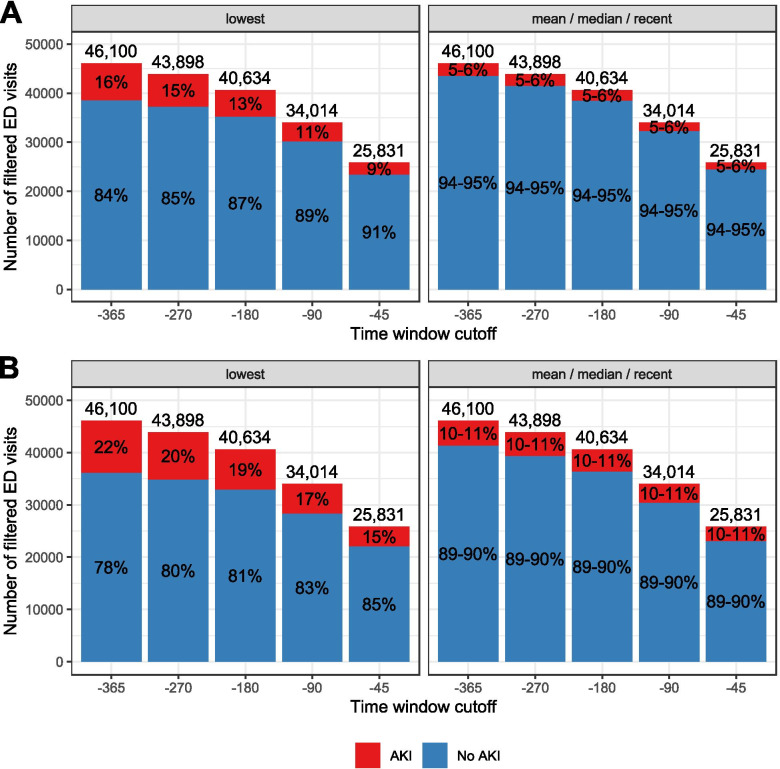
AKI prevalence at emergency department calculated with multiple baseline definitions and markers (serum creatinine and eGFR). For each baseline time window a surrogate marker was selected from the specified baseline time window with one of the four baseline values. Depending on the availability of measurements, emergency department visits were filtered in each baseline time window described as cutoff. Panel **A** shows the relative increase of ≥1.5 serum creatinine criterion to baseline and panel **B** shows relative decrease of < 25% in eGFR

Next, we evaluated the effect of reducing the baseline time window from − 365-7 to − 45/− 7 for both surrogate makers (criteria 4 and 7). Adjusting the time window from − 365/− 7 to − 45/− 7 reduced the number of visits with available baseline values from 46,100 to 25,831 (44.0% decrease). Apart from the lowest baseline value, AKI prevalence remained the same for the mean, median and most recent baseline values when reducing the time window for both SCr and eGFR criteria. The SCr criterion with the lowest baseline value in combination with the − 45/− 7 days time window led to a reduction in AKI prevalence from 15.8 to 9.0%, whereas the eGFR criterion with the lowest baseline value showed a reduction from 21.4 to 14.5% (Fig. 3).

### RIFLE guidelines yield the highest AKI prevalence

We compared the RIFLE, AKIN and KDIGO guidelines by applying the guideline-related criteria in combination with the different baseline definitions. To include all ED visits, we used the − 365/− 7 baseline time window for the two criteria without definition for baseline time window (criteria 4 and 7). Regardless of the used baseline definition (lowest, recent, median or mean), the AKI prevalence was different between the guidelines. Between the three guidelines, we found a maximum difference in AKI prevalence of 23.1% (range between 5.9 and 24.0%) (Table 3). Again, the lowest value resulted in the highest AKI prevalence (RIFLE; 24.0%, AKIN; 7.7%, KDIGO; 16.2%).

**Table 3:** AKI prevalence at emergency department based on different guidelines

Guideline	Baseline value	N visits	AKI (%)	AKI hospital admissions (%)	AKI KDIGO stage (%)
Rifle	Lowest	47,373	11,354 (24.0%)	8524 (75.1%)	0: 3677 (32.4%)
					1: 4903 (43.2%)
					2: 1872 (16.5)
					3: 902 (7.9)
	Mean	47,373	5476 (11.6%)	4478 (81.8%)	0: 2711 (49.5%)
					1: 1889 (34.5%)
					2: 567 (10.4%)
					3: 309 (5.6%)
	Median	47,373	5493 (11.6%)	4505 (82.0%)	0: 2424 (44.1%)
					1: 2082 (37.9%)
					2: 644 (11.7%)
					3: 343 (6.2%)
	Most recent	47,373	5234 (11.0%)	4340 (82.9%)	0: 2369 (45.3%)
					1: 1935 (37.0%)
					2: 601 (11.5%)
					3: 329 (6.3%)
AKIN^a^	Lowest	3322	256 (7.7%)	231 (90.2%)	1: 139 (54.3%)
					2: 62 (24.2%)
					3: 55 (21.5%)
	Mean	3322	248 (7.5%)	223 (89.9%)	1: 192 (77.4%)
					2: 30 (12.1%)
					3: 26 (10.5%)
	Median	3322	247 (7.4%)	222 (89.9%)	1: 189 (76.5%)
					2: 30 (12.1%)
					3: 28 (11.3%)
	Most recent	3322	247 (7.4%)	223 (90.3%)	1: 197 (79.8%)
					2: 23 (9.3%)
					3: 27 (10.9%)
KDIGO	Lowest	47,373	7694 (16.2%)	6023 (78.3%)	1: 4920 (63.9%)
					2: 1872 (24.3%)
					3: 902 (11.7%)
	Mean	47,373	2798 (5.9%)	2430 (86.8%)	1: 1922 (68.7%)
					2: 567 (20.3%)
					3: 309 (11.0%)
	Median	47,373	3105 (6.6%)	2664 (85.8%)	1: 2118 (68.2%)
					2: 644 (20.7%)
					3: 343 (11.0%)
	Most recent	47,373	2914 (6.2%)	2550 (87.5%)	1: 1984 (68.1%)
					2: 601 (20.6%)
					3: 329 (11.3%)

Prevalence shown as percentages between brackets was calculated on visits with at least one baseline value and a serum creatinine measurement at emergency department visit (*N* = 47,373). The most recent baseline definition was used in combination with the −365/−7 days time window to diagnose AKI. Number and percentage of hospital admission and KDIGO stage are shown for patients with AKI according to the guideline

^a^As the AKIN criteria only evaluate the 48 h prior to ED visit, only visits with measurements within this period were used to calculate the AKI prevalence (*N* = 3322)

## Discussion

In this retrospective study, we evaluated the effect of different baseline definitions on AKI diagnosis at the ED, using prevalence as an outcome. Interestingly, the AKI prevalence varied considerably under different guidelines ranging between 5.7 and 23.6%. Our study shows that selecting the SCr-BL with the *lowest* baseline definition resulted in the highest AKI prevalence. Furthermore, we also found that expanding the baseline time window length resulted in a higher AKI prevalence for the lowest baseline definition. Moreover, applying the RIFLE guidelines criteria for AKI resulted in the highest prevalence. Our study shows that definition of baseline has important implications for the diagnosis of AKI at the ED.

To our knowledge, this is the first study that performed an in-depth analysis on a large cohort providing insights in the effect of applying various definitions, criteria and guidelines to diagnose AKI at ED. Studies on AKI epidemiology in hospitalized patients have been well described with reported prevalence rates that vary between < 1–66% [21]. However, studies in the ED population are scarce. Our results are in line with the sparse literature. A previous study in the ED applied the AKIN criteria, corresponding to our − 2/0 baseline time window analysis, and found a prevalence of 6.4%, which is similar to the AKI prevalence of 7.8% in our study [22]. Another study found an AKI ED prevalence of 5.5% by applying the KDIGO criteria with a baseline defined 1 year prior ED visit [23]. However, of the studies that compared RIFLE, AKIN and KDIGO for the definition of AKI, none of them investigated the different definitions of baseline with different baseline time-windows [24, 25].

Apart from diagnosing AKI, baseline SCr is also necessary to evaluate the deterioration of renal function in AKI and to follow the extent of recovery after an AKI event. The methods to estimate baseline SCr in literature include admission SCr, lowest value prior to admission, minimum SCr value during hospital admission or a calculation using the MDRD equation in patients without baseline [26]. The definition for baseline SCr is of great importance, since this can significantly affect the assessment of the AKI prevalence and of the associated mortality risk and renal outcomes. Taking the SCr at admission has been shown to be unrepresentative as baseline, since this value could be influenced by the ongoing disease [27]. On the other hand, using the lowest baseline definition may overestimate AKI prevalence, as shown in this study. As a result, several studies have over- or underestimated AKI diagnosis using different baseline definitions in different populations [28]. Also, in our study, baseline definition in terms of baseline value and time window show major consequences on AKI prevalence at ED. For each baseline time window, the most recent value might be the most accurate representation of the patient’s premorbid renal function to determine AKI, and may reduce over-estimation of AKI diagnoses. Moreover, instead of evaluating all baseline definitions, only evaluating the most recent baseline time window may reduce the number of false positive diagnoses. In contrast, we show that including all guideline criteria as well as all baseline time windows increases the number of AKI cases, which may lead to over-estimation of AKI diagnoses at the ED.

Our study has several strengths. We used a large dataset with a well-documented population. This allowed us to study the AKI prevalence at the ED using real-world data reflecting current clinical care at the ED of several years. Furthermore, the use of a relational database, which continuously stores laboratory and clinical data for every patient, ensures maximum completeness and integrity of the data, which are often problems associated with retrospective data analyses.

This study also has several limitations. The current study is a retrospective single center study in an academic center, therefore these results may not be generalizable to other patient populations. Additional studies are necessary to determine the extent to which the definition of baseline affects AKI prevalence in other clinical settings. Furthermore, in the current literature, baseline SCr is defined as a measurement in a healthy person. By including ED visits of patients with at least one SCr value in their clinical history we may have introduced a selection towards sicker patients as they had their kidney function assessed previously. Defining criteria for baseline SCr that reflects kidney function in a stable healthy condition would be ideally to compare the SCr value with the value at admission to the ED, and also for application of CDS [13]. However, as an academic hospital the majority of the patients with a blood test at ED receive a SCr test during routine visit, thereby reducing this bias in our analyses. In addition, the values we included were the available SCr values used by clinicians, providing a better reflection of clinical practice and the current AKI prevalence for diseased patients to explore the challenges of AKI diagnosis at ED. Lastly, half of the ED visits either did not have a SCr-BL and/or a SCr-ED (results not shown). For this reason, we may have excluded patients with an elevated SCr during ED visit who may have had AKI. As a consequence, the computed rate of AKI diagnosis may not reflect the true AKI incidence at our ED. AKI incidence at ED estimated with the discussed guidelines may result in an underestimation of the true AKI incidence.

To compare the guidelines in terms of baseline definition, we adapted the criteria by selecting one measurement from the baseline time window. However, the AKIN criteria evaluate all SCr measurements from the 48 h prior to ED. Comparing all SCr measurements from this time window with the SCr-ED may have resulted in a higher AKI prevalence. However, regardless of baseline definition, AKIN AKI prevalence remained the same indicating that the majority of the visits only had one SCr measurement available, thereby reducing this bias in our analyses. As only a small proportion (*N* = 3322, 7.0%) of all visits had at least one SCr measurement in the 48 h prior to ED visit, computing the prevalence on all patients result in a lower AKI prevalence at ED. Moreover, we did not exclude patients who revisited our center during the study inclusion time window. Recurrent patients may have more complications that might be associated with a higher chance of AKI. As a result, this may have influenced the AKI prevalence. However, evaluating the AKI prevalence at the ED requires the complete ED population, including repetitive visits of not-critically ill patients.

Multiple additions can be made to the guidelines to improve diagnosis. For example, the CKD-EPI formula may provide inaccurate estimations of kidney function in patients as this formula was developed on healthy patients of which the concentration of SCr was in steady-state. As a consequence, GFR estimates may be over- or underestimated that may affect AKI diagnosis. Dynamic eGFR formulas such as suggested by Chen et al. (2013) may provide a better estimate of GFR [29]. Moreover, the addition of novel biomarkers for AKI diagnosis, such as neutrophil gelatinase-associated lipocalin (NGAL), cycle arrest biomarkers, tissue inhibitor metalloproteinase-2 (TIMP-2) and insulin-like growth factor-binding protein (IGFBP7), may be added in the future to the guidelines to improve AKI diagnosis [30].

Importantly, our data provide evidence that researchers and developers of CDS systems and machine learning algorithms based on different baseline definitions for AKI criteria could account for major changes on the diagnosis of AKI when their research is implemented in the ED setting. The use of different definitions for baseline may thus lead to delayed recognition and underestimation of AKI and affect the clinical course and initiation of therapy in the early stages of AKI. As more hospitals implement AKI guidelines for automated AKI diagnosis, being familiar with the consequences of ambiguous baseline definitions is of upmost importance. As large routine care datasets facilitate retrospective research and prospective implementation of clinical decision support systems, researchers as well as CDS developers should carefully weigh the pros and cons of different definitions, criteria and guidelines in terms of false positive or false negative diagnoses in multidisciplinary teams to tailor the definition of AKI to their needs [31].

## Conclusions

In conclusion, this study shows that ambiguous definitions for baseline can have major consequences on the AKI diagnosis in patients presenting at the ED. Incorrect definition of baseline may result in misdiagnosis of AKI patients at the ED with suboptimal decisions for treatment and medication as result. Clinicians, as well as researchers and developers of automatic diagnostic tools such as clinical decision support systems should take these considerations into account when aiming to diagnose AKI in clinical and research settings.

## Supplementary Information

**Additional file 1: Supplementary data Table 1.** Frequency table of the number of emergency department visits of all 20,488 included patients. **Supplementary data Table 2.** Mean and standard deviation of baseline (BL) serum creatinine (SCr) and eGFR for each baseline time window and baseline value. **Supplementary data Table 3.** AKI prevalence for each of the seven criteria combined with each baseline value. **Supplemental Figure 1.** Boxplots of delta serum creatinine (SCr) between the selected baseline SCr value and the SCr measurement at emergency department visit, for each baseline definition. **Supplemental Figure 2.** Boxplots of delta glomerular filtration rate (eGFR) between the selected baseline eGFR value and the eGFR measurement at emergency department visit, for each baseline definition.

## Data Availability

The data underlying this article cannot be shared publicly due to ethical and privacy reasons for the privacy of individuals that participated in the study. The data will be shared on reasonable request to the corresponding author.

## Abbreviations

AKI: Acute kidney injury
ED: Emergency department
SCr: Serum creatinine
SCr-ED: Serum creatinine concentration at emergency department visit
SCr-BL: Baseline serum creatinine concentration
EHR: Electronic health records
CDS: Clinical decision support
eGFR: Estimated Glomerular Filtration Rate
UMCU: University Medical Center Utrecht
UPOD: Utrecht Patient Orientated Database
