# Supplementary material for: Ambiguous definitions for baseline serum creatinine affect acute kidney diagnosis at the emergency department
Source: BMC Nephrol. 2021 Nov 8;22:371. doi: 10.1186/s12882-021-02581-x (PMC8573871; doi:10.1186/s12882-021-02581-x)
Supplement: Supplementary file 1 — Additional file 1: Supplementary data Table 1. Frequency table of the number of emergency department visits of all 20,488 included patients. Supplementary data Table 2. Mean and standard deviation of baseline (BL) serum creatinine (SCr) and eGFR for each baseline time window and baseline value. Supplementary data Table 3. AKI prevalence for each of the seven criteria combined with each baseline value. Supplemental Figure 1. Boxplots of delta serum creatinine (SCr) between the selected baseline SCr value and the SCr measurement at emergency department visit, for each baseline definition. Supplemental Figure 2. Boxplots of delta glomerular filtration rate (eGFR) between the selected baseline eGFR value and the eGFR measurement at emergency department visit, for each baseline definition. [file 12882_2021_2581_MOESM1_ESM.docx]

## Supplemental Material

**Table 1:** frequency table of the number of emergency department visits of all 20488 included patients.

**Table 2:** mean and standard deviation of baseline (BL) serum creatinine (SCr) and eGFR for each baseline time window and baseline value.

**Table 3:** AKI prevalence for each of the seven criteria combined with each baseline value

**Figure 1**: boxplots of delta serum creatinine (SCr) between the selected baseline SCr value and the SCr measurement at emergency department visit, for each baseline definition.

**Figure 2:** boxplots of delta glomerular filtration rate (eGFR) between the selected baseline eGFR value and the eGFR measurement at emergency department visit, for each baseline definition.

**Supplementary data Table 1: frequency table of the number of emergency department visits of all 20488 included patients.**

| Emergency department visits per patient | Count (%) |
| --- | --- |
| 1  2  3  4  5  >5 | 11256 (54.9%)  3923 (19.1%)  2014 (9.8%)  1128 (5.5%)  619 (3.0%)  1548 (7.6%) |

**Supplementary data Table 2: mean and standard deviation of baseline SCr and eGFR for each time window and value.**

| Time window (days) | Baseline value | N | Mean SCr baseline in µmol/L | Mean eGFR baseline |
| --- | --- | --- | --- | --- |
| -365/-7 | lowest | 46,100 | 85.7±75.8 | 86.8±29.8 |
| -365/-7 | mean | 46,100 | 104.6±105.8 | 78.5±29.4 |
| -365/-7 | median | 46,100 | 102.6±105.6 | 78.9±29.9 |
| -365/-7 | most recent | 46,100 | 102.0±107.1 | 79.2±30.1 |
| -7/0 | lowest | 10,554 | 105.5±101.5 | 78.1±31.2 |
| -7/0 | mean | 10,554 | 108.5±106.9 | 77.1±31.1 |
| -7/0 | median | 10,554 | 108.5±104.7 | 77.1±31.2 |
| -7/0 | most recent | 10,554 | 107.4±104.7 | 77.3±31.1 |

**Supplementary data Table 3: AKI prevalence for each of the seven criteria combined with each baseline value.** Prevalence was computed on the number of visits in each time window.

| Criterion | Option | N visits | AKI (%) |
| --- | --- | --- | --- |
| Rise to >= 1.5 times SCr in 365 days | Lowest | 46,100 | 7,304 (15.8%) |
|  | Most recent | 46,100 | 2,572 (5.6%) |
|  | Median | 46,100 | 2,417 (5.9%) |
|  | Mean | 46,100 | 2,417 (5.9%) |
| Decrease >25% eGFR in 365 days | Lowest | 46,100 | 9,879 (21.4%) |
|  | Most recent | 46,100 | 4,682 (10.2%) |
|  | Median | 46,100 | 4,970 (10.8%) |
|  | Mean | 46,100 | 5,020 (10.9%) |
| Rise to >= 1.5 times SCr in 7 days | Lowest | 10,554 | 489 (4.6%) |
|  | Most recent | 10,554 | 401 (3.8%) |
|  | Median | 10,554 | 390 (3.7%) |
|  | Mean | 10,554 | 388 (3.7%) |
| Decrease >25% eGFR in 7 days | Lowest | 10,554 | 932 (8.8%) |
|  | Most recent | 10,554 | 803 (7.6%) |
|  | Median | 10,554 | 790 (7.5%) |
|  | Mean | 10,554 | 800 (7.6%) |

**Supplemental Figure 1: boxplots of delta SCr between SCr-BL and SCr-ED, for each baseline definition.** Number of emergency department visits in each baseline time window are shown above the boxplots.

**Supplemental Figure 2: boxplots of delta eGFR between the selected eGFR-BL and eGFR-ED, for each baseline definition.** Number of emergency department visits in each baseline time window are shown above the boxplots.
